# Small molecules targeting Progranulin as potential therapeutics for Frontotemporal Dementia (FTD) and other dementias

**DOI:** 10.1002/alz.087605

**Published:** 2025-01-09

**Authors:** Suman Chatterjee, Joshua Lorenz‐Guertin, Junriz O. Delos Santos, Mohna Bandyopadhyay, Monica Tomaszewski, Kari Dilley, Emma Hartman, Scott Sneddon

**Affiliations:** ^1^ Sharp Therapeutics, Pittsburgh, PA USA

## Abstract

**Background:**

Progranulin (*GRN*) plays a critical role in familial frontotemporal dementia (fFTD), where *GRN* haploinsufficiency leads to reduction in PGRN levels in the brain, resulting in degeneration of neurons in the frontal lobe of brain responsible for personality, language, and behavior. FTD is the most common dementia in people under 60. Sortilin (*Sort1*), expressed by neurons, endocytoses, and delivers PGRN rapidly to lysosomes for degradation. To compensate the lack of PGRN, therapeutic strategies to increase extracellular PGRN have been pursued to address fFTD with no approved treatments currently available.

**Methods:**

We performed an SH‐SY5Y cell‐based assay to identify small molecules that reduce PGRN endocytosis. Validation of lead candidates was performed by the same assay in mouse neuroblastoma cell line, N2a and rat neurons. This was followed by medicinal chemistry for structure optimization to improve the potency and stability of the compounds. Compounds with better *in vitro* potency and stability were then further optimized through an iterative series of pharmacokinetic (PK), and pharmacodynamic (PD) assessments.

**Results:**

Sharp has discovered a series of structurally related compounds with potent EC_50_ values in blocking Sortilin‐mediated PGRN endocytosis in multiple cell lines from different species allowing levels of progranulin to increase almost two‐fold. In addition, our lead compound has been shown to increase extracellular PGRN levels in a dose dependent manner. Finally, intraperitoneal administration of our lead compound(s) to mice resulted in a dose and exposure‐dependent increase in PGRN measured in brain tissue extracts and plasma. Lead compounds were further characterized for CNS drug‐like properties including PK, metabolism, brain penetration, and are currently being evaluated in selectivity, and safety assays.

**Conclusions:**

Our approach generated a series of novel small molecules, especially our lead candidates that show favorable drug‐like characteristics *in vitro* and *in vivo* and are well‐suited to be clinically evaluated for the treatment of fFTD.

